# Measurement of Bisphenol A Diglycidyl Ether (BADGE), BADGE derivatives, and Bisphenol F Diglycidyl Ether (BFDGE) in Japanese infants with NICU hospitalization history

**DOI:** 10.1186/s12887-023-04493-1

**Published:** 2024-01-08

**Authors:** Mami Kuwamura, Kentaro Tanaka, Atsuto Onoda, Kentaro Taki, Chihaya Koriyama, Kyoko Kitagawa, Toshihiro Kawamoto, Mayumi Tsuji

**Affiliations:** 1 Department of Environmental Health, University of Occupational and Environmental Health, 1-1, Iseigaoka, Yahatanishi-ku Kitakyushu, Fukuoka, 807-8555 Japan; 2 Department of Pediatrics, University of Occupational and Environmental Health, 1-1, Iseigaoka, Yahatanishi-ku Kitakyushu, Fukuoka, 807-8555 Japan; 3 Division of Neonatology, Center for Maternal-Neonatal Care, Nagoya University Hospital, 65 Tsurumai-Cho Showa-Ku, 466-8550 Nagoya, Japan; 4 Department of Toxicology and Health Science, Faculty of Pharmaceutical Sciences, Sanyo-Onoda City University, 1-1-1 Daigaku-dori Sanyo-Onoda, Yamaguchi, 756-0884 Japan; 5 Division for Medical Research Engineering, Nagoya University Graduate School of Medicine, 65 Tsurumai-Cho Showa-Ku, 466-8550 Nagoya, Japan; 6 Department of Epidemiology and Preventive Medicine, Kagoshima University Graduate School of Medical and Dental Sciences, 8-35-1, Sakuragaoka, Kagoshima, 890-8544 Japan

**Keywords:** Bisphenol A diglycidyl ether, Bisphenol F diglycidyl ether, Neonatal exposure, Medical equipment

## Abstract

**Background:**

Bisphenol A diglycidyl ether (BADGE) and Bisphenol F diglycidyl ether (BFDGE) are used in medical devices, such as intravenous sets, syringes, and catheters. Several studies have reported that these compounds are endocrine disruptors, cytotoxic, and genotoxic, raising concerns about their adverse effects on infants, in a stage of remarkable growth and development. The present study aimed to measure the serum concentrations of BADGE, derivatives of BADGE, and BFDGE in infants and examine the factors that influence them.

**Methods:**

Ten infants admitted to the neonatal intensive care unit (NICU) were enrolled in the present study. Blood samples from each infant and questionnaires from their mothers were collected twice, at 1–2 months and 7 months of age. BADGE, BADGE·H_2_O, BADGE·2H_2_O, and BFDGE were quantified using liquid chromatography-tandem mass spectrometry (LC-MS/MS).

**Results:**

Serum BADGE·2H_2_O was identified in all infants, at both 1–2 months (2.30–157.58 ng/ml) and 7 months of age (0.86–122.85 ng/ml). One of the two infants who received invasive ventilation showed a substantially increased BADGE·2H_2_O concentration. There was no significant difference in BADGE·2H_2_O concentrations at 7 months of age between the group that ate commercial baby food at least ≥ 1 time per week and the group that did not.

**Conclusions:**

BADGE·2H_2_O was detected in the serum of all infants with a history of NICU hospitalization. Future studies are needed to determine the source of BADGE exposure and investigate its effects on infant development.

**Supplementary Information:**

The online version contains supplementary material available at 10.1186/s12887-023-04493-1.

## Background

Epoxy and vinyl chloride resins are widely used for the inner coating of canned and retort pouch foods. Bisphenol A diglycidyl ether (BADGE) is synthesized when epoxy resins are produced via the condensation of Bisphenol A (BPA) and epichlorohydrin [1]. Similarly, the reaction of phenol formaldehyde resin with epichlorohydrin produces bisphenol F diglycidyl ether (BFDGE). During storage, food coatings can interact with aqueous and acidic foods, leading to the formation of hydrolysis derivatives, such as BADGE·2H_2_O, BADGE·H_2_O, BFDGE·2H_2_O, and BFDGE·H_2_O, that may migrate into the food. Furthermore, BADGE and BFDGE can be added to remove the hydrochloric acid produced during thermal coating, which could result in the formation of chlorine derivatives [2]. Migration of BADGE, BFDGE, and their derivatives into canned foods and beverages has been reported in several countries. For instance, derivatives of BADGE have been detected in canned vegetables and mixed dishes in France, [3] while BADGE and derivatives of BADGE have been found in canned fish, vegetables, and sauces in Japan [4]. Both BADGE and BFDGE have been detected in infant formulas in Canada [5]. Therefore, food is regarded as a major source of exposure to BADGE and BFDGE [6]. BADGE, BFDGE, and their derivatives have been detected in adult human body fluids and tissues [6–8]. Furthermore, BADGE is also used in paints, adhesives, floorings, clothing, textiles, and furniture coatings [9, 10]. Infants characteristically put anything they can reach into their mouths, increasing their potential exposure to BADGE and its toxic effects.

The capacity of BADGE and its derivatives to disrupt endocrine function and induce cytotoxicity and genotoxicity raises significant concerns regarding their safe use [11–13]. Infants expressing BADGE-specific immunoglobulin G (IgG) showed high food-specific immunoglobulin E (IgE) levels, suggesting that BADGE affects the immune system [14]. Furthermore, BFDGE has been implicated in cytotoxic, mutagenic, and genotoxic effects [15]. Accordingly, the commission of the European Communities has set a limit on their migration into food, up to 9 mg/kg for the sum of BADGE and its hydrolyzed derivatives (BADGE·H_2_O, BADGE·2H_2_O) and 1 mg/kg for the sum of BADGE formed as reaction products with HCl (BADGE·HCl, BADGE·2HCl, BADGE·H_2_O·HCl) [16]. The Commission has also prohibited the use or presence of BFDGE. However, there are no regulations regarding the use of BADGEs or BFDGE in medical devices.

In addition to everyday applications, various medical devices, such as intravenous (IV) sets, syringes, and catheters, are made of plastics, such as BPA-based polycarbonate, and epoxy resins are used as adhesives. Because BADGE is synthesized during the production of epoxy resins, medical devices can be a source of exposure to BADGE. Neonates admitted to the neonatal intensive care unit (NICU) require several medical devices for life support, including incubators, ventilators, IV sets, gastroesophageal tubes, and urinary catheters. A previous study on neonates admitted to the NICU reported that the increased use of medical devices was associated with high levels of BPA in their urine, regardless of their oral exposure to BPA [17]. High levels of urinary BPA have also been observed in patients in pediatric intensive care units, where medical devices are frequently used [18, 19]. However, there are no reports on the association between the use of medical devices and BADGE concentrations.

The purpose of the present study was to measure serum levels of BADGE, derivatives of BADGE, and BFDGE in infants with a history of hospitalization in the NICU and evaluate their association with medical devices and the living environment. Derivatives of BFDGE were not included owing to a lack of established standards and detection methods.

## Methods

### Study design

Sixteen neonatal patients admitted to the NICU of the University of Occupational and Environmental Health, Japan between November 2017 and November 2019, whose parents provided consent to participate, were enrolled in the study. Their reasons for hospitalization included premature birth, low birth weight, transient tachypnea of the newborn (TTN), neonatal respiratory distress syndrome (RDS), or hyperbilirubinemia. Exclusion criteria were infants with inherited metabolic diseases, chromosomal abnormalities, or congenital malformations. We collected blood samples from the infants twice: once at 1–2 months to assess the effects of medical equipment used in the NICU, and a second time at the seven month medical checkup, to assess the effects of living at home. The first samples were collected during the blood tests needed for treatment in the NICU and for evaluation after discharge. Japanese infants typically begin eating baby food at around 6 months of age, consequently parents become more concerned about food allergies at this time. Therefore, the second set of samples were collected when blood tests were performed on infants whose parents requested testing for food allergies at the seven month examination. Approximately 3–5 mL blood was collected with a butterfly needle connected to a syringe, and 2 ml of sample was used for this study. Six infants were excluded because we were unable to obtain serum samples at seven months of age. Consequently, 10 neonates were ultimately included.

Information on infants during NICU admission was collected from medical records and included gestational age, height, weight, feeding type (breast milk or formula) and method (tube or bottle) of nutrition, as well as days of medical equipment use (peripheral infusion, central venous catheter, gastroesophageal tube, ventilator, nasal intermittent positive pressure ventilator, high-flow nasal cannula, and incubator). Nasal intermittent positive pressure ventilation (NIPPV) provides respiratory support via nasal prongs [20]. All the baby bottles used in the NICU were made of glass.

Information on infants was collected from questionnaires completed by the mothers at the time of serum sample collection. The questionnaire included questions on nutrition type and method (breastfeeding or plastic or glass bottles), children’s toy materials, and baby food intake. In addition, to ascertain epoxy resin exposure to family members, the question, “Does anyone in your family use the following in their jobs or hobbies?: paints, adhesives, printing presses, or imported furniture " was included. We obtained information about the medical history of mothers during pregnancy.

### Detection of BADGE

#### Chemicals

Chemicals, including BADGE (B519500) and BFDGE (B519540), BADGE·2H_2_O (15,137), and BADGE·H_2_O (73,417), were procured from FUJIFILM Wako Chemicals (Osaka, Japan) and Sigma-Aldrich (St. Louis, MO, USA) for analytical standards. Internal standards, BADGE-D6 (TRC-B519502) and BFDGE-^13^C_12_ (CIL-CLM-9867), were obtained from LGC Standards (Teddington, United Kingdom). These substances were of liquid chromatography-mass spectrometry (LC-MS) or high-performance liquid chromatography (HPLC) grade, with molecular structures shown in Figure S1. Milli-Q water and LC-MS grade methanol (MeOH) from an ultrapure water system (Barnstead International, Dubuque, IA, U.S.) were used. Serum samples were stored below − 30 °C, and solutions for calibration and working standards were prepared by dilution of stock solutions in MeOH.

Calibrators and quality controls were spiked in MeOH from the stock solutions, resulting in concentrations of 1.0, 0.1, 0.01, and 0.001 ng/µL. A working solution of the internal standard (0.01 ng/µL BADGE-D6) in MeOH was prepared. Calibrators were spiked with the spiking solutions to achieve a final concentration range of 0.05–100 ng/mL in 200 µL of water. Quality control samples for each run were freshly prepared by adding a spiking solution to achieve concentrations of 1.0, 10, and 50 ng/mL in 200 µL of blank serum matrixes.

#### Sample preparation

Samples were prepared following previously described procedure [7, 21]. In brief, 200 µL of serum was transferred to a glass tube, and 0.3 ng of BADGE-D_6_ and BFDGE-^13^C_12_ internal standards were added to blanks and samples before extraction. Extraction was performed using a 1:1 (v/v) mixture of ethyl acetate and hexane (2 mL total volume). After vortexing and centrifugation at 3000 rpm for 10 min, the supernatant was dried with a TurboVap LV evaporator under nitrogen at 35 °C. The remaining substances were dissolved in 200 µL of the initial mobile phase, and a 10-µL aliquot was injected into the LC-MS/MS system.

#### Detection and measurement conditions

The Shimadzu Prominence-i HPLC (Shimadzu Co., Kyoto, Japan) and QTRAP® 6500 mass spectrometer (AB Sciex Pte. Ltd., Framingham, MA, USA) were utilized for target chemical separation and quantification. L-column2 ODS (1.5 × 150 mm and a particle size of 3 μm) from the Chemicals Evaluation and Research Institute (Tokyo, Japan) was employed with a gradient elution of 5% methanol (A) and 95% water (B), both containing 5 mM ammonium acetate. The flow rate was maintained at 0.10 ml/min, staring with a 70:30 (A/B) ratio for 5 min and increasing to 100% B over 20 min. Mass spectrometry operated in positive electrospray ionization mode, with capillary and vaporizer temperatures set at 500 °C, an ion spray voltage of 4500 V, and the nitrogen sheath and auxiliary gases were set to 40 arbitrary units. Ion pairs m/z 358 → 191, m/z 376 → 209, m/z 394 → 209.0, m/z 330 → 163.0, m/z 364 → 197.0 and m/z 342 → 169 were used to quantify BADGE, BADGE·H_2_O, BADGE·2H_2_O, BFDGE, BADGE-D6, and BFDGE-13C2, with optimized collision energies at 19, 19, 21, 17, 19 and 17 eV, respectively. MultiQuant software (ver. 2.2.1, AB Sciex Pte. Ltd.) was employed for LC-MS/MS data analysis.

#### Validation methods and data analysis

Validation methods and data analysis followed procedures from a previous study [21]. In the internal standard method, a calibration curve for each analyte was constructed from the ratio of analyte response to internal standard response in measured standard solutions. All validation criteria, including reproducibility and accuracy, were determined based this ratio. Quantification used the pre-extraction matrix-spiked calibration curve. Replicate analysis (*N* = 3) involved measuring standard solutions with consistent sample preparation and instrumental analysis. Matrix-matched calibration standards and solvent calibration standards verified instrumental calibration and correlation coefficients. Accuracy and precision, demonstrated as relative standard deviation (RSD), were assessed through inter-day precision and recovery experiments (n = 3). Accuracy and precision of each concentration ranged from 81.6 to 103.2% and 1.41–7.65% RSD (Table S1). The matrix effect was evaluated by observing the LC-MS/MS response changes due to co-elution of endogenous matrix components comparing a pure standard solvent solution injection.

The excretion speed per date (ESD) was calculated using the equation:

$$ESD=\frac{\left({S}_{2}-{S}_{1}\right)}{\left(C{D}_{2}-C{D}_{1}\right)}$$

Where S_1_ refers to the concentration (ng/mL) of the first sample, S_2_ to the concentration (ng/mL) of the second sample, where CD_1_ refers to the first collection date and CD_2_ to their second collection date.

### Statistical analyses

Data were analyzed by using STATA version 14.0. Data normality was evaluated using the Shapiro–Wilk test. Numerical variables showing non-normal distributions are presented as median values. Concentrations of the first and second BADGE derivatives were compared using the Wilcoxon signed-rank test.

As BADGE has been detected in retort foods, including baby food, BADGE concentrations were compared by categorizing them according to the frequency of commercial baby food intake [3]. The Mann–Whitney U test was used to compare the concentration of BADGE derivatives between the two groups. Statistical significance was set at *P* < 0.05.

## Results

We summarized the characteristics of the participating infants at the time of first and second serum sample collection (Table 1). The gestational age ranged from 30 to 38 weeks; 90% of the infants were preterm (< 38 weeks), and 80% had low birthweight (< 2500 g). The length of their hospital stays ranged from 5 to 62 days. Among the participants, the first serum samples were collected from three infants while still in the NICU (infant number 1–3). For the remaining participants, the first serum samples were collected between 8- and 39-days post-discharge from the NICU. Infants 2 and 3 required respiratory support through endotracheal intubation, and their first serum samples were collected 53 and 31 days after extubation, respectively.

**Table 1:** Characteristics of study participants

									1st collection				2nd collection	
Infant number	Sex	Weeks of gestation	Delivery method	Birth weight (g)	Birth length (cm)	Cause of hospitalization	Maternal pregnancy complications	Hospitalization period (days)	Age (days)	Days after extubation	Days after NICU discharge	Infant weight (kg)	Age (days)	Infant weight (kg)
1	Boy	30	Cesarean	1513	43	Preterm, low birth weight, TTN	Threatened premature delivery	62	56	-	0	2.59	261	6.92
2	Boy	30	Vaginal	1749	41	Preterm, low birth weight, RDS	Nothing	59	56	53	0	2.80	212	5.76
3	Girl	32	Cesarean	1804	42	Preterm, low birth weight, RDS	Gestational diabetes mellitus	35	34	31	0	2.44	229	7.41
4	Boy	33	Cesarean	2105	47	Preterm, low birth weight	Threatened premature delivery	24	32	-	8	2.84	214	8.13
5	Boy	34	Vaginal	2042	44	Preterm, low birth weight	Type 2 diabetes mellitus, pregnancy-induced hypertension	25	37	-	12	3.07	219	8.70
6	Boy	34	Vaginal	2796	49	Preterm, TTN	Threatened premature delivery	20	46	-	26	3.99	228	9.05
7	Boy	35	Vaginal	2302	46	Preterm, low birth weight	Premature rupture of membrane	19	47	-	28	4.07	220	8.12
8	Boy	35	Vaginal	2382	45	Preterm	Nothing	24	63	-	39	4.53	222	6.85
9	Boy	35	Cesarean	2718	50	Preterm, hyperbilirubinemia	Premature rupture of membrane	28	65	-	37	3.45	254	7.17
10	Girl	38	Cesarean	3210	48	Hypoglycemia	GDM	5	33	-	28	4.21	215	8.20

The details of nutritional intake and home environment obtained from the questionnaire were described (Table 2). Families of infants 3, 4, 5, and 6 answered that they use products containing epoxy resin at the time of the first or second questionnaire. All those families used printing presses, but only one of those families (that of infant 5) also used adhesives.

**Table 2:** Feeding details and household environment of infants

	1st collection					2nd collection					
Infant number	Nutriton type	Formula volume (ml/kg/day)	Feeding bottle material	Family use of products containing epoxy resins	Infant toy material	Nutrition type	Frequency of weaning food (/day)	Frequency of commercial baby food intake	Family use of products containing epoxy resins	Infant toy material	Infant tableware material
1	Breast milk & formula	77.2	Glass	Nothing	Not used	Formula & weaning food	1	Everyday	Nothing	Plastic, fabric	Plastic
2	Breast milk & formula	178.6	Glass	Nothing	Not used	Formula & weaning food	1	Nothing	Nothing	Plastic, fabric	Plastic, glass
3	Breast milk & formula	10.3	Glass	Nothing	Not used	Formula & weaning food	2	1~3/month	Printing presses	Plastic, fabric, wood	Plastic
4	Breast milk & formula	61.7	Glass	Printing presses	Wood	Formula & weaning food	2	Everyday	Nothing	Plastic, wood	Plastic, glass
5	Formula only	214.8	Plastic	Nothing	Plastic, fabric	Formula & weaning food	2	1~3/month	Adhesives, printing presses	Plastic, fabric	Plastic, glass
6	Breast milk only	0.0	Not use	Printing presses	Plastic, fabric, wood	Breast milk & Formula & weaning food	2	Nothing	Printing presses	Plastic, fabric, wood	Plastic, glass, metal
7	Breast milk & formula	78.6	Glass	Nothing	Unknown	Formula & weaning food	1	1-3/week	Nothing	Plastic, fabric, wood	Plastic
8	Breast milk & formula	171.0	Plastic, Glass	Nothing	Plastic, wood	Formula & weaning food	3	4-6/week	Nothing	Wood	Metal, other
9	Breast milk & formula	23.2	Plastic, Glass	Nothing	Plastic, fabric	Breast milk & weaning food	1	1-3/month	Nothing	Plastic, fabric	Plastic
10	Breast milk & formula	166.3	Glass	Nothing	Fabric	Breast milk & Formula & weaning food	2	1-3/week	Nothing	Plastic, fabric	Plastic

The duration of medical device use during the NICU admission showed that all infants received peripheral or central venous infusions (Table 3). Infants 1, 2, 3, and 6 were provided with respiratory support due to respiratory impairment, and infants 2 and 3 underwent invasive ventilation due to respiratory distress syndrome. Infants with lower gestational age were exposed to more medical devices for longer durations.

**Table 3:** Duration of medical device use (days) on infants in the NICU

Infant number	Peripheral infusion	Peripherally inserted central catheter	Nasogastric tube	Nasal intermittent positive pressure ventilation	High-flow nasal cannula	Ventilator	Neonatal care incubator
1	0	14	48	23	24	0	40
2	0	11	38	13	16	2	32
3	0	7	17	4	0	2	16
4	0	9	14	0	0	0	9
5	0	8	10	0	0	0	10
6	0	8	6	3	0	0	4
7	5	0	7	0	6	0	8
8	0	5	6	0	0	0	8
9	8	0	3	0	0	0	4
10	4	0	0	0	0	0	3

In all serum samples, only BADGE·2H_2_O was quantified, while BADGE·H_2_O and BADGE were at their lower limit of detection (LLOD, < 0.09 ng/mL), and BFDGE was at its lower limit of quantitation (LLOQ, < 0.39 ng/mL, Table 4). The highest BADGE·2H_2_O concentration in the first serum sample was in infant number 3 (157.58 ng/mL), one of the infants who received invasive ventilation. Concentrations of BADGE·2H_2_O tended to be lower in the second serum sample collected than in the first (*P* = 0.0593). However, the BADGE·2H_2_O concentration in the second sample collected from infant 5 was higher than in the first sample (122.85 ng/mL). The median excretion speed of BADGE·2H_2_O per date was − 0.06 ng/mL/day (range: -0.80–0.47) (Table 4).

**Table 4:** Concentrations of BADGEs and BFDGE in serum samples and excretion speed

	Concentrations (ng/mL) of 1st serum sample				Concentrations (ng/mL) of 2nd serum sample				ESD (ng/mL/day)
Infant number	BADGE·2H_2_O	BADGE·H_2_O	BADGE	BFDGE	BADGE·2H_2_O	BADGE·H_2_O	BADGE	BFDGE	BADGE·2H_2_O
1	25.24	LLOD	LLOD	LLOQ	0.86	LLOD	LLOD	LLOQ	-0.12
2	2.43	LLOD	LLOD	LLOQ	1.04	LLOD	LLOD	LLOQ	-0.01
3	157.58	LLOD	LLOD	LLOQ	2.56	LLOD	LLOD	LLOQ	-0.80
4	2.54	LLOD	LLOD	LLOQ	1.73	LLOD	LLOD	LLOQ	0.00
5	36.78	LLOD	LLOD	LLOQ	122.85	LLOD	LLOD	LLOQ	0.47
6	2.30	LLOD	LLOD	LLOQ	1.52	LLOD	LLOD	LLOQ	0.00
7	32.29	LLOD	LLOD	LLOQ	3.78	LLOD	LLOD	LLOQ	-0.16
8	11.02	LLOD	LLOD	LLOQ	7.04	LLOD	LLOD	LLOQ	-0.03
9	21.03	LLOD	LLOD	LLOQ	2.48	LLOD	LLOD	LLOQ	-0.10
10	25.60	LLOD	LLOD	LLOQ	2.45	LLOD	LLOD	LLOQ	-0.13
median (min-max)	23.13 (2.30–157.58)				2.47 (0.86–122.85)				-0.06 (-0.80–0.47)

*ESD *Excretion speed per date, *LLOD *Lower limit of detection, <0.09 ng/mL. *LLOQ* lower limit of quantitation, <0.39 ng/mL

The concentrations of BADGE·2H_2_O at the second sample collection grouped by frequency of commercial baby food consumption were compared (Table 5). There was no significant difference in BADGE·2H_2_O concentrations in the samples obtained at the second collection between the group that consumed commercial baby food less than once per week and the group that consumed it more often than once per week (*P* = 0.9168).

**Table 5:** Differences in BADGE·2H_2_O concentration by frequency of commercial baby food

Frequency of commercial baby food	BADGE·2H_2_O at 2nd time of collection
<1/week (infant number 2,3,5,6,9)	1.04–122.85
≥1/week (infant number 1,4,7,8,10)	0.86–7.04

## Discussion

The objective of this study was to assess the levels of BADGE, derivatives of BADGE, and BFDGE in infants previously hospitalized in the NICU and examine their potential correlation with medical devices and living conditions. In the present study, the serum levels of BADGE, derivatives of BADGE, and BFDGE were measured twice, at approximately 1–2 months of age and again at approximately 7 months of age, in 10 infants with a history of NICU admission. Only BADGE·2H_2_O was present at levels above the threshold value of the testing method at both sampling times, whereas BADGE·H_2_O, BADGE, and BFDGE were not. There have been no previous reports measuring the serum concentrations of BADGE, derivatives of BADGE, and BFDGE in infants. Additionally, there have been no previous reports evaluating the exposure to BADGE via medical devices. Consequently, this is the first report examining the relationship between medical device use and living environment at home and serum BADGE·2H_2_O concentrations in infants with a history of NICU hospitalization.

While no reports of measuring BADGEs (BADGE and its derivatives) or BFDGEs (BFDGE and its derivatives) in infants have been published, urinary BPA has been measured [16]. Urinary BPA levels were significantly higher in infants in the NICU who used four or more medical devices within three days than in infants who used three or fewer medical devices [17]. Although breast milk and formula BPA concentrations were also measured, no significant differences were found, and the infants’ urinary BPA concentrations did not differ according to the feeding method. Furthermore, urinary BPA levels were significantly high in pediatric intensive care unit patients who were endotracheally intubated or who underwent hemodialysis [18]. These reports suggested that invasive medical procedures increase chemical exposure from medical devices. In the present study, the first sample in infants 1–3 was collected while they were in the NICU. Therefore, the concentration of the first samples of those three infants reflected the chemical exposure in the NICU. Of the three, infant 3 had the highest BADGE·2H_2_O concentration, followed by infant 1, while infant 2 had the lowest BADGE·2H_2_O concentration. All three infants were fed breast milk and formula, and no correlation was observed between the amount of formula and BADGE·2H_2_O concentration. Infants 2 and 3 were both on ventilators for two days; however, the BADGE·2H_2_O concentration was remarkably high for only infant 3. The first serum sample from infant 2 was collected 53 days after extubation, whereas the sample from infant 3 was collected 31 days after extubation, which is a discrepancy in timing that may have contributed to the difference in BADGE·2H_2_O concentrations observed. These findings may suggest that invasive ventilation is associated with increased serum BADGE concentrations, similar to a previously reported association between BPA and invasive ventilation [17]. Although infant 3 had been extubated for 31 days prior to the sampling, his BADGE·2H_2_O concentration was still clearly higher than that of the other infants and may have been even higher during endotracheal intubation management. There are no previous reports on the daily excretion rate of BADGE·2H_2_O. The daily excretion rate calculated in the present study was affected by new or sustained exposures between the first and second sample collection. Therefore, it is important to note that this excretion rate does not simply represent the rate at which BADGE·2H_2_O is excreted from the body.

There was no significant difference, but nine of the ten infants had lower BADGE·2H_2_O concentrations in the second sample than in the first, which may be due to the potential influence of the insertion of various medical devices during the NICU admission and immaturity of metabolism and excretion. In humans, BADGE is complexly hydrolyzed, oxidized, and conjugated to produce several derivatives, which are excreted in urine and feces [22]. Epoxide hydrolases, working primarily in the liver, enzymatically hydrolyze BADGE to produce the first by-product, BADGE·H_2_O. Further hydrolysis by the same enzyme produces BADGE·2H_2_O, which is further oxidized by monooxygenase to other oxidation byproducts [22]. Many cytochrome P450s that function as monooxygenases have low or no activity at birth, and their activity gradually increases over the first 3 months of life [23]. These suggest that delayed biotransformation of BADGE·2H_2_O in infants at 1–2 months of age occurs and may induce its accumulation. In addition, it is well known that renal function in infants is immature. Glomerular filtration rate (GFR) at birth is 19.6 ml/min per 1.73 m^2^ and gradually increases to 59.4 ml/min per 1.73 m^2^ by 4 weeks of age [24]. Subsequently, GFR increases until about 2 years of age, when it reaches the adult values [25]. Therefore, the lower excretion of BADGE·2H_2_O in urine in early infancy might be one of the causes of higher serum concentration in the first sample.

Several reports have assessed the exposure to BADGEs and BFDGEs in both adults and children. The urinary levels of BADGE and its derivatives (BADGE·H_2_O, BADGE·2H_2_O, BADGE·H_2_O·HCl) observed in 57 adult volunteers from the U.S. and China, as well as 70 Chinese children, indicated that BADGE·2H_2_O was the predominant substance detected among them [26]. Furthermore, BADGE·2H_2_O was detected with the highest frequency, exceeding 90%, among the nine BADGE derivatives (BADGE·H_2_O, BADGE·2H_2_O, BADGE·H_2_O·HCl, BADGE·2HCl, BADGE, BFDGE·2H_2_O, BFDGE·H_2_O, BFDGE·2HCl, and BFDGE) in the serum and urine of 181 Chinese children and adolescents [27]. Hydrolysis of BADGE·H_2_O to BADGE·2H_2_O is rapid, but oxidation of BADGE·2H_2_O to other byproducts by monooxygenase is slow [22]. Therefore, BADGE·2H_2_O possibly accumulates in the body and is the predominant BADGE metabolite. The serum and urinary concentrations of BADGE·2H_2_O in 181 Chinese children and adolescents were up to 38.441 ng/mL and 8.902 ng/mL, respectively, indicating that concentrations of BADGE·2H_2_O are high in human blood [27]. Compared to serum levels of BADGE·2H_2_O in Chinese children and adolescents, those of some infants in the present study were markedly higher (157.58 ng/mL in infant 3 and 122.85 ng/mL in infant 5). These previous reports indicate a high detection rate of BADGE·2H_2_O, which was similar to the results of the present study; however, unlike in previous reports, other BADGEs and BFDGE were not detected in infants, which may be due to differences in the sources of exposure and metabolism.

Furthermore, urinary levels of BADGE·2HCl, BADGE·2H_2_O, BADGE·H_2_O, BADGE·HCl, and BFDGE·2H_2_O were negatively correlated with age, suggesting that younger individuals may have a higher risk of exposure [27]. Sealants, paints, adhesives, furniture coatings, textile fillers, packaging materials, dental fillers, and coatings inside cans all contain BADGE [28]. Moreover, BADGE and its derivatives have also been detected in the air and indoor dust [29–31]. Infants who live closer to the floor and have higher respiratory rates than adults are more prone to ingesting dust particles. Only one infant in our study, infant 5, had a markedly higher concentration of BADGE·2H_2_O at the second time of sample collection than at the first. The family of infant 5 was the only one who indicated in the second questionnaire that they used adhesives. It is unclear who in the family was using the adhesives and how, but it may be related to the significantly high BADGE·2H_2_O concentrations in infant 5. Since BADGE·2H_2_O is formed by the hydrolysis of BADGE in the body [22], it is expected that the infant’s BADGE·2H_2_O would be elevated due to exposure to some BADGE.

Several studies demonstrated the toxicity of BADGE and its derivatives. For instance, BADGE·2HCl and BFDGE·2HCl exhibit antiandrogenic effects by binding to the androgen receptors in vitro; [13]. BADGE·2H_2_O and BADGE 2Cl induce breast cancer cell proliferation via an estrogenic mechanism, without binding to estrogen receptors [32]. Moreover, BADGE and BFDGE were found to induce morphological changes and cell detachment from the substrate in Caco-2 cells derived from human intestinal epithelial cells [33]. As infants are vulnerable to damage due to being in a state of remarkably rapid growth and development, it is necessary to assess the impact of BADGE and BFDGE on children. Based on our results and previous findings, we suggest that younger children face a greater risk of exposure to BADGE·2H_2_O. Future studies should increase the number of cases and determine the effects of BADGE·2H_2_O on infants.

The absence of a control group is a limitation of the present study, making it unclear whether the medical equipment used during NICU admission caused the BADGE exposure. Further, it is of considerable importance that this study was able to investigate the relationship between the differences in medical device use and BADGE·2H_2_O concentrations in infants with the same background history of NICU admission. There is uncertainty regarding the human placental transfer of BADGE or its derivatives, with a report that BADGE·2H_2_O was detected in only 1 sample out of 14 umbilical cord blood samples [7]. The placental transfer of BADGE·2H_2_O may have affected its concentrations in infants, but this was unknown because maternal BADGE·2H_2_O concentrations were not measured in this study. In addition, the concentration of BADGE in breast milk and formula and that released from baby bottles was not measured; thus, oral exposure was not evaluated. Similarly, BADGEs in needles, syringes, spits, and other medical devices used for blood collection have not yet been evaluated. Finally, the present study did not investigate whether commercial baby food packaging is indeed a source of exposure to BADGEs because information about the packaging material of commercial baby food was not available.

## Conclusion

In the present study, BADGE·2H_2_O was detected in infants with a history of NICU hospitalization. Medical equipment used during NICU hospitalization might be a source of BADGE exposure. However, due to the small sample size and absence of controls, a causal relationship is not clear. One infant showed consistently high levels of BADGE·2H_2_O even after discharge from the hospital, suggesting additional exposure to BADGE in the home environment. Further studies with larger sample sizes are required to better understand the extent of BADGE exposure and its effects on infants.

### Supplementary Information

**Additional file 1: Figure S1.** Molecular structures of BADGE, BADGE·H_2_O, BADGE·2H_2_O, BADGE-D6, BFDGE, and BFDGE-^13^C_12_

**Additional file 2: Table S1.** Accuracy and precision of target chemicals

## Data Availability

The datasets analyzed during the current study are available from the corresponding author on reasonable request. However, to protect personal identification, the data about individuals are not shared.

## Abbreviations

BADGE: Bisphenol A Diglycidyl Ether
BFDGE: Bisphenol F Diglycidyl Ether
BPA: Bisphenol A
HPLC: High-performance liquid chromatography
IgE: Immunoglobulin E
IgG: Immunoglobulin G
LLOD: Lower limit of detection
LLOQ: Lower limit of quantitation
MeOH: Methanol
NIPPV: Nasal positive pressure ventilation
NICU: Neonatal intensive care unit
SRM: Signal reaction monitoring
