# Supplementary material for: Measurement of Bisphenol A Diglycidyl Ether (BADGE), BADGE derivatives, and Bisphenol F Diglycidyl Ether (BFDGE) in Japanese infants with NICU hospitalization history
Source: BMC Pediatr. 2024 Jan 8;24:26. doi: 10.1186/s12887-023-04493-1 (PMC10773092; doi:10.1186/s12887-023-04493-1)

Figure S1 Molecular structures of BADGE, BADGE・H_2O, BADGE・$2H_2O$, BADGE-D6, BFDGE, and BFDGE-$^{13}C_{12}$

Bisphenol A Diglycidyl Ether (BADGE)

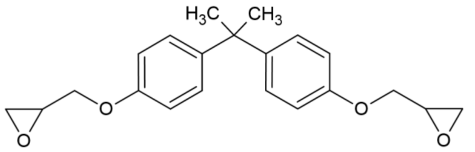

Bisphenol A (2,3-dihydroxypropyl) glycidyl ether (BADGE・H_2O)

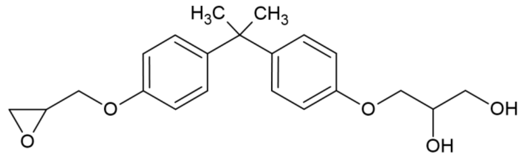

Bisphenol A bis(2,3-dihydroxypropyl) ether (BADGE・$2H_2O$)

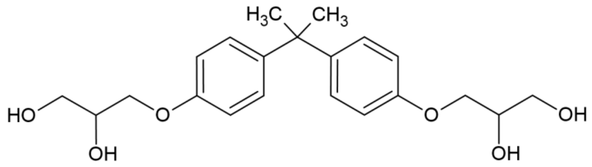

Bisphenol A-d6 Diglycidyl Ether (BADGE-D6)

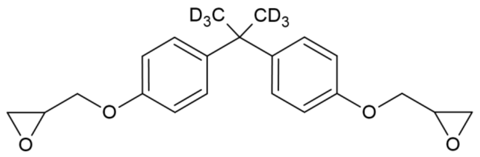

Bisphenol F diglycidyl ether (BFDGE)

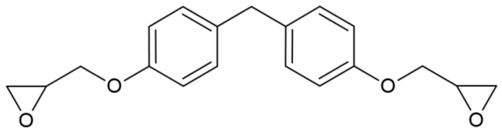

Bisphenol F diglycidyl ether (RING-$^{13}C_{12}$) (BFDGE-$^{13}C_{12}$)

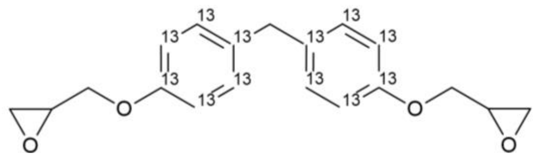

Supplement: Supplementary file 1 — Additional file 1: Figure S1. Molecular structures of BADGE, BADGE·H2O, BADGE·2H2O, BADGE-D6, BFDGE, and BFDGE-13C12 [file 12887_2023_4493_MOESM1_ESM.pdf]
