# Supplementary material for: Measurement of Bisphenol A Diglycidyl Ether (BADGE), BADGE derivatives, and Bisphenol F Diglycidyl Ether (BFDGE) in Japanese infants with NICU hospitalization history
Source: BMC Pediatr. 2024 Jan 8;24:26. doi: 10.1186/s12887-023-04493-1 (PMC10773092; doi:10.1186/s12887-023-04493-1)
Supplement: Supplementary file 2 — Additional file 2: Table S1. Accuracy and precision of target chemicals [file 12887_2023_4493_MOESM2_ESM.pdf]

Table S1 Accuracy and precision of target chemicals

**Compounds**	**Conc. (ng/mL)**	**Precision (% RSD)**	**Accuracy (%)**
BADGE	0.5	2.63	83.9
	1	1.50	91.7
	10	2.79	102.7
BADGE·H_2O	0.5	1.41	90.6
	1	2.48	96.6
	10	2.50	100.8
BADGE·$2H_2O$	0.5	7.65	83.5
	1	2.73	91.3
	10	5.28	102.2
BFDGE	0.5	3.34	81.6
	1	2.92	91.1
	10	1.42	103.2

n=3

RSD: Relative standard deviation.
